# Pharmacological targets and therapeutic mechanisms of Arabic gum in treating diabetic wounds: insights from network pharmacology and experimental validation

**DOI:** 10.3389/fphar.2025.1528880

**Published:** 2025-02-20

**Authors:** Langjie Chai, Danyang Chen, Lili Ye, Pan Peng, Haijie Wang, Nouf Al Saleh, Nader S. Al-Kenani, Jia Guo, Qianqian Li, Liang Guo

**Affiliations:** ^1^ Department of Plastic Surgery, Zhongnan Hospital of Wuhan University, Wuhan, China; ^2^ Smart Hybrid Materials Laboratory (SHMs), Division of Physical Science and Engineering, King Abdullah University of Science and Technology (KAUST) Thuwal, Thuwal, Saudi Arabia; ^3^ N. Al Saleh Bioengineering Institute, Health Sector, King Abdul Aziz City for Science and Technology (KACST), Riyadh, Saudi Arabia; ^4^ Department of Orthopedic, Prince Sultan Bin Abdulaziz Humanitarian City, Riyadh, Saudi Arabia

**Keywords:** diabetic wound healing, Arabic gum, network pharmacology, advanced glycation end products, AGEs-NF-κB-NLRP3 axis

## Abstract

**Background and objectives:**

On account of the long-term inflammatory microenvironment, diabetic wounds are challenging to heal in which advanced glycation end products are considered important factors hindering the healing of diabetic wounds. Gum Arabic has demonstrated significant potential in the treatment of various diseases owing to its anti-inflammatory and antioxidant properties. Nonetheless, there is still insufficient research on the role of Arabic gum in facilitating diabetic wounds healing and its mechanisms. This study aims to investigate the pharmacological targets and therapeutic mechanisms of Arabic Gum on diabetic wound healing by adopting network pharmacology, molecular docking, and experimental validation.

**Methods:**

Key active components of Arabic Gum and disease targets were identified through network pharmacology and bioinformatics. GO/KEGG enrichment was performed to identify critical pathways. Cytoscape and AutoDock were used for targets prediction and molecular docking validation. *In vitro*, Transwell assay and tube formation assay were performed to evaluate the effect of Arabic Gum on human fibroblasts migration and human umbilical vein endothelial cells angiogenesis. Western blotting analyzed Pro-caspase-1, ASC, NLRP3 and NF-κB pathway-related proteins. *In vivo*, a full-thickness diabetic wound model was established. Histological changes were assessed via H&E and Masson’s staining, oxidative stress levels through DHE staining, inflammation levels with IL-1β, CD68 and CD206 staining, angiogenesis and cell proliferation levels were assessed by CD31 and Ki67 staining. The levels of pathway-related proteins were analyzed by NLRP3 and Phospho-NF-κB P65 staining.

**Results:**

Network pharmacology analysis identified key targets, encompassing HSP90AA1, STAT3, and PRKCB, involved in the AGEs-NF-κB-NLRP3 signaling axis. Molecular docking demonstrated strong binding affinity between AG components and these targets. *In vitro*, AG lessened AGEs-induced activation of the NLRP3 inflammasome via modulation of the NF-κB pathway and reinforced cell migration and angiogenesis. *In vivo*, AG-treated diabetic wounds exhibited accelerated healing, with augmented collagen deposition, lowered oxidative stress and inflammation, and strengthened cell migration and angiogenesis. AG promotes diabetic wound healing by modulating the AGEs-NF-κB-NLRP3 axis, exerting anti-inflammatory, antioxidant, pro-angiogenic, and cell-proliferative effects.

**Conclusion:**

This study provides new insights into diabetic wound repair and suggests that AG is a promising therapeutic agent for improving diabetic wound healing.

## Graphical Abstract

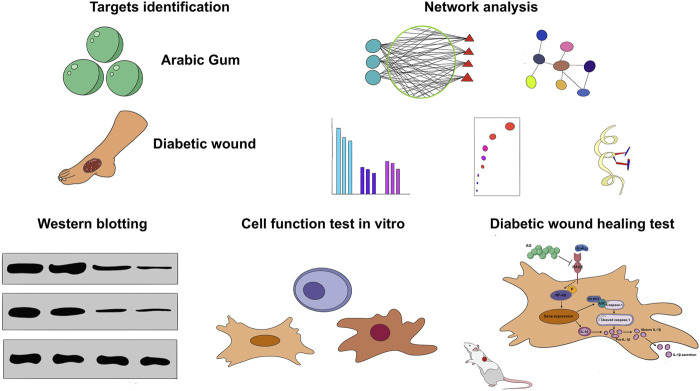

## 1 Introduction

For the time being, over 537 million adults (aged between 20 and 79) across the globe are suffering from diabetes, which is a figure anticipated to heighten to 643 million by the year of 2030 (Ong et al., 2023). Chronic wounds bound up with diabetes represent one of the most prevalent complications of the disease, with approximately 1 in 6 individuals with diabetes will experience such wounds. Diabetic wounds are linked to a significantly elevated risk of limb amputation and increased mortality rates. Research indicates that individuals with diabetic wounds face a 50%–68% higher risk of mortality over a five-year period (Jiang et al., 2023). Presently, existing treatment modalities for diabetic wounds, including debridement and wound offloading, have demonstrated inadequate efficacy, underscoring the necessity for the development of more effective management strategies for these wounds (McDermott et al., 2023; Wang et al., 2024).

At both the microvascular and macrovascular levels, complications are primary contributors to illness and death among individuals with type 2 diabetes. Notwithstanding the complexity to comprehend the pathophysiology of type 2 diabetes, cutting-edge glycation end products (AGEs) are regarded as significant factors that push ahead the advancement of diabetes and the emergence of its related complications (Lee et al., 2022). It is believed that diabetic complications primarily arise from uncontrolled hyperglycemia, which brings about the formation of AGEs.

It’s noteworthy that the formation of endogenous AGEs primarily takes place via a complex, multi-step process of glycation referred to as the Maillard reaction. As illustrated by growing evidence, in the context of chronic diabetes, sustained hyperglycemia gives rise to heightened levels of AGEs in the blood. These AGEs, by binding to their receptor known as RAGE, trigger a cascade of signaling events. Such events encompass the generation of reactive oxygen species (ROS), calcification, and the formation of thrombi in the arterial walls. An overproduction of ROS can give rise to oxidative stress and inflammatory reactions, which brings about detrimental influences on the process of wound healing (Dunnill et al., 2017; Khalid et al., 2022; Chiu et al., 2023). For this reason, products formed through advanced glycation contribute to the aging of blood vessels and associated damage.

Nuclear factor kappa-B (NF-κB) functions as a pivotal transcription factor, initiating the activation of numerous genes integral to the modulation of inflammatory responses and is ubiquitous across virtually all cell types (Aggarwal et al., 2006). Upon exposure to dissimilar extracellular stimuli, rapid phosphorylation of NF-κB occurs, which in turn modulates gene transcription. AGEs, a category of external substances affecting the activation of NF-κB, can reinforce and prolong signaling pathways, thereby giving rise to inflammatory responses (Li J.-S. et al., 2022; Shu et al., 2023). Aside from that, the inflammasome that contains the nucleotide-binding domain (NBD), leucine-rich repeat (LRR), and pyrin domain (PYD)-bearing protein 3 (NLRP3) serves as a crucial mediator within innate immune responses. Comprising the NOD-like receptor NLRP3, the adaptor protein ASC, and caspase-1, the NLRP3 inflammasome conducts a paramount role (Fu and Wu, 2023; Zheng et al., 2023). Both the NLRP3 inflammasome and NF-κB are crucial in the inflammatory progression of several diseases (Wang et al., 2018; Zhao et al., 2019; Peng et al., 2020), leading to a prolonged state of chronic inflammation that delays the wound healing process in diabetes.

Arabic Gum (AG), a natural and thick exudate obtained from the branches of Acacia seyal and Acacia senegal, is extensively acknowledged by the U.S. Food and Drug Administration (FDA) as a safe source of dietary fiber (Al-Jubori et al., 2023). Numerous experimental investigations have underlined the potential merits of AG in clinical applications. As reported by Ahmed et al. (2022) and his team, the antioxidant and anti-inflammatory effects of AG might counteract oxidative harm, inflammation, and cell death stemmed from exposure to aflatoxin B1 in animal subjects. Abu-Serie et al. (2021) and her colleagues evidently illustrated that AG could reduce systemic oxidative stress and necroinflammatory responses resulting from CCl4 administration. The anti-inflammatory and antioxidant properties of AG exert beneficial effects on diabetic wounds featured by chronic inflammation.

Network pharmacology integrates systems biology with network informatics, which favorably provides profound insights into molecular mechanisms from an all-round standpoint. It functions as both a theoretical basis and a technical resource for contemporary drug development, which not only accelerates the process of identifying active compounds, but also clarifies drug effectiveness. This methodology corresponds with the traits of drugs that are multi-component, multi-targeted, and exhibit synergistic effects (Nogales et al., 2022; Shang et al., 2023). As a consequence, by utilizing a blend of network pharmacology, molecular docking, and experimental validation, we delved further into the active ingredients, possible targets, and molecular mechanisms of Arabic Gum in addressing diabetic chronic wounds. Initial validation was performed via cellular and animal experiments, offering instructive guidance for the subsequent development and application of Arabic Gum in reinforcing the healing of diabetic chronic wounds.

## 2 Materials and methods

### 2.1 Network pharmacology-based analysis

#### 2.1.1 Collection and screening of active chemical composition in Arabic gum

In this research, a search was performed on PubMed utilizing “Arabic Gum” as our primary keyword to determine the active components of AG (Ashour et al., 2022; Afoakwah et al., 2023). The molecular designations were submitted to the public chemical database PubChem (https://pubchem.ncbi.nlm.nih.gov/) to acquire the molecular representations in Canonical SMILES format (Kim et al., 2023). The molecular structures in.mol 2 format and Canonical SMILES expressions were then imported into the SwissTargetPrediction database (http://www.swisstargetprediction.ch/) (Daina et al., 2019). Active targets were recognized grounded in criteria of norm fit >0.9 and the top 15 rankings, separately. Subsequent to the consolidation of the results and the elimination of duplicates, the potential target names were standardized with the UniProt database (https://www.uniprot.org/) (UniProt Consortium, 2018). Ultimately, the information was merged with data from existing literature.

#### 2.1.2 Collection of the targets of diabetic chronic wound

A keyword search was performed utilizing “diabetic wound” in the GeneCards database (https://www.genecards.org/) (Rebhan et al., 1997), the OMIM database (https://www.omim.org/) (Amberger et al., 2015), and the DisGeNET database (https://www.disgenet.org/) to discover pertinent targets (Piñero et al., 2020). The outcomes were consolidated, and duplicate records were eliminated.

#### 2.1.3 Construction the network of the drug-components-targets-pathways-disease

The main components and potential targets of AG from Section 2.1.1, the potential targets for diabetic chronic wounds from Section 2.1.2, and the KEGG pathways bound up with diabetic wounds analyzed in Section 2.1.5 were imported into Cytoscape 3.8.2 to construct a drug-components-targets-pathways-disease network.

#### 2.1.4 Construction of PPI network of common targets of AG and diabetic wound

The identification of the overlap between the targets of AG active components and those bound up with chronic wounds in diabetes was conducted by employing Venny 2.1 (https://bioinfogp.cnb.csic.es/tools/venny/). Afterwards, the overlapping targets were uploaded to the STRING database (https://cn.string-db.org/) (Szklarczyk et al., 2023), where “*Homo sapiens*” was selected for the species, and a confidence score greater than 0.700 was specified, while disconnecting nodes were hidden. The resulting data was then exported and visualized by adopting Cytoscape 3.8.2. For the topological analysis of the resultant PPI network, the CentiScaPe 2.2 plugin in Cytoscape 3.8.2 was utilized accordingly (Franz et al., 2023). Core targets were determined by filtering the network through parameters exceeding the calculated values of betweenness centrality, closeness centrality, and degree.

#### 2.1.5 GO analysis and KEGG pathway enrichment analysis

The primary objectives of AG and chronic wounds associated with diabetes were uploaded to the DAVID database (https://david.ncifcrf.gov/) (Sherman et al., 2022), selecting “*H. sapiens*” as the species. The outcomes were subjected to filtering and analysis, utilizing a significance threshold of P < 0.05.

#### 2.1.6 Molecular docking

The primary targets identified in Section 2.1.4 were prioritized on the basis of their degree values, which was arranged in an order from high to low. Afterwards, the three highest-ranking key targets and their relevant AG active components were chosen for molecular docking analysis. The chemical structures of these active components of AG, sourced from the TCMSP database (Ru et al., 2014), were analyzed by employing PyMOL 2.6 and AutoDock 1.5.7, whereas the protein crystal structures predominantly originated from the PDB database (Nawaz et al., 2023). Calculations of binding energies were performed, and the resulting data were visualized through PyMOL 2.6.

### 2.2 *In vitro* experiment

#### 2.2.1 Materials

Arabic Gum (AG) were purchased from Shanghai Aladdin Biochemical Technology Co., Ltd. AGE-BSA (AGEs) were purchased from Biogradetech. All other chemical reagents are of analytical grade.

#### 2.2.2 Cell culture and treatment

Mouse macrophage cells (RAWs) (Thermo Fisher Scientific), human skin fibroblasts (HSFs) (Fenghui Biotechnology) and human umbilical vein endothelial cells (HUVECs) (Thermo Fisher Scientific) were cultured in DMEM medium (Thermo Fisher Scientific), DMEM/F12 medium (Thermo Fisher Scientific) and 1640 medium (Thermo Fisher Scientific) supplemented with 10% fetal bovine serum (FBS) and 1% penicillin-streptomycin in a 5% CO_2_ incubator at 37°C. In accordance with the experimental group design, they were divided into three groups: the control group (NC group), the AGEs group, and the AGEs + AG group. The pretreatment was carried out 24 h in advance, followed by the next experiments.

#### 2.2.3 Cell viability assay

Human skin fibroblasts (HSFs) were seeded into 96-well plates at a concentration of 1 × 10^4^ cells per well. Following exposure to varying AG concentrations (25, 50, 100, and 200 mg/mL) for periods of 24 and 72 h, 10 μL of CCK-8 solution (Beyotime, Shanghai, China) was introduced to each well. After a 1-h incubation at 37°C, the absorbance of each well was recorded at 450 nm. The cell viability in response to AG was determined by utilizing the following formula:

$$Cell\;viability\;\left(\%\right)=\frac{A_{S}-A_{0}}{A_{C}-A_{0}}\times 100\%$$

where *A*_*S*_ represents the absorbance of cells with AG, *A*_*C*_ symbolizes the absorbance of cells and *A*_*0*_ refers to the absorbance of cell media.

The dose response curve was plotted to compute the 50% (IC 50) concentration of AG inhibiting cell growth.

#### 2.2.4 *In vitro* blood compatibility assay

To comprehensively evaluate blood biocompatibility, a method was utilized in a systematic manner, where rat citrated blood was mixed with saline in a ratio of 5: 4. A volume of 1 mL from this saline-diluted blood was incorporated into the AG solution, which was prepared at a concentration of 25 mg/mL, and subsequently incubated for 1 h at 37°C. By contrast, red blood cells treated with normal saline served as the control group. After a 5-min centrifugation at 1,000 RPM, 100 μL of the supernatant was transferred into a 96-well plate, and the optical density was measured at 540 nm by employing a microplate reader. The formula below was employed to compute the percentage of hemolysis:

$$Hemolysis\;\left(\%\right)=\frac{OD_{S}-OD_{B}}{OD_{C}-OD_{B}}\times 100\%$$

where *OD*_*S*_ *, OD*_*B*_ and *OD*_*C*_ are the optical density of sample, blank (Normal saline treated with RBCs as negative control) and Control (Triton-X treated with RBCs as positive control) severally.

#### 2.2.5 Transwell assay

The upper chamber was inoculated with HSFs at a density of 2 × 10^4^ cells per well. Afterwards, the cells were treated in the lower chamber for 24 h under a range of group conditions, then fixed by utilizing formaldehyde, stained with crystal violet, and observed through an inverted fluorescence microscope for photography. On top of that, cell scratch assays were conducted under diverse grouping scenarios, with images captured by adopting an inverted microscope (IX73, Olympus) after 24 and 48 h.

#### 2.2.6 Tube formation assay

In an effort to assess the angiogenic potential of AG, a tube formation assay was conducted accordingly. First and foremost, 100 μL of Matrigel (Corning) was placed in a 96-well plate and allowed to gel at 37°C for 1 h. Afterwards, human umbilical vein endothelial cells (HUVECs) were seeded onto the Matrigel at a density of 3 × 10^4^ cells per well, with 100 μL of media that included various groups: AGEs group, AGEs + AG group, and a control group with media only. Subsequent to a 6-h incubation, the development of capillary-like structures was observed by adopting an inverted microscope (IX73, Olympus). The tube networks were quantitatively analyzed with the angiogenesis analyzer in ImageJ software (NIH), measuring parameters such as the number of junctions and total segment length.

#### 2.2.7 Western blotting

The lysing of cultured cells was carried out by utilizing RIPA buffer (Beyotime, China) and protease inhibitor (PMSF, Biosharp, China). A 20 g protein sample was separated via a 10% SDS-PAGE technique, subsequently transferred to polyvinylidene difluoride membranes (Millipore Sigma). To block the membranes, 5% non-fat milk was applied at room temperature for 1 h. Following this, immunoblotting was performed on the separated proteins, which were probed with anti-Pro Caspase-1 + p10 + p12 Rabbit mAb (ABclonal, China; #A25308, 1:2,000), anti-ASC/TMS1 Rabbit mAb (22,046, 1:6000), anti-NLRP3 (ABclonal, China; #A24294, 1:500), anti-NF-κB p65 mAb (Proteintech, China; #66535-1-lg, 1:1,000), and anti-Phospho-NF-κB p65 rAb (Proteintech, China; #82335-1-RR, 1:2000) overnight at 4 °C. The following day, the membranes underwent rinsing for 10 min with Tris-buffered saline containing Tween 20, followed by incubation at room temperature with a peroxidase-conjugated secondary antibody (Abcam, United Kingdom; ab205718, 1:10,000) (Biosharp, China; BL001A, 1:5,000) for 1 h. Protein bands were visualized by utilizing strengthened chemiluminescence detection. Quantitative analysis of the immunoreactive bands was performed by employing ImageJ software. Three technical and three experimental replicates were conducted.

### 2.3 *In vivo* experiment

#### 2.3.1 Preparation of type 2 diabetic rat model

All animal experimentation conducted in this research adhered to the guidelines sanctioned by the Institutional Animal Care and Use Committee at the Hubei Provincial Center for Disease Control and Prevention (IACUC Number: 202,320,189). Type I diabetic rat model was established in rats in line with previously reported protocols (Huang et al., 2024). A group of male Sprague-Dawley (SD) rats weighing approximately 250 ± 25 g underwent a 1-week acclimatization period. Following an 18-h fasting period, the rats were administered with injection of streptozotocin (STZ) at a dose of 75 mg/kg daily for three consecutive days. Afterwards, the blood glucose levels of the rats were monitored every 3 days over a span of 3 weeks. Rats exhibiting a stable glucose level exceeding 16.6 mM were classified as having successfully developed a type 2 diabetes model. The animals were then randomly assigned for additional experiments.

#### 2.3.2 Diabetic wound healing test

Animals were anesthetized by sevoflurane inhalation, and the dorsal of rats were shaved or depilated. A circular biopsy punch with a diameter of 15 mm was employed to induce full-thick wounds on the dorsal of rats. Diabetic wounds were received dissimilar treatments, comprising 1) Blank control group (negative group); 2) *Comfeel* ^®^ hydrocolloid dressing group (HCD group, No.1 positive control group); 3) YOUZHI ^®^ medical chitosan dressing group (MCH group, No.2 positive control group); 4) Acacia Gum Group (AG Group), which topically uses four diverse materials to cover the wound, next, Tegaderm™ Company then (3M, USA) covers the wound area and changes it every 3 days. Wounds were photographed at dissimilar time points during healing process. Afterwards, wound areas in each group were measured by ImageJ software (NIH, United States).

#### 2.3.3 Histological analysis

On days 3, 6, and 19 following the injury, we collected and fixed skin tissues around the wounds for histological examination. With an aim to assess epidermal regeneration and wound inflammation, we conducted Hematoxylin & Eosin (H&E) staining. Collagen deposition within the wound bed was assessed by adopting Masson’s trichrome staining. Aside from that, we employed DHE and antibodies against IL-1β, CD68, CD206, CD31, Ki67, NLRP3, and Phospho-NF-κB P65 to analyze tissue oxidative stress, pro-inflammatory markers, macrophage polarization, angiogenesis, cell proliferation, inflammasome activity, and NF-κB pathway activation, severally.

#### 2.3.4 Statistical analysis

Each experiment included a minimum of three independent trials. The data are presented as mean ± standard deviation (SD). Statistical analyses were performed by employing one-way analysis of variance (ANOVA) to compare multiple groups, and graphs were generated by utilizing GraphPad Prism 9.0 (San Diego, CA, United States). P-values were classified as follows: *p < 0.05, **p < 0.01, ***p < 0.001, and ****p < 0.0001. A P-value of <0.05 is regarded as statistically significant, whereas a P-value >0.05 is considered not significant (ns).

## 3 Result

### 3.1 Network pharmacology-based analysis

Through a PubMed literature search (Afoakwah et al., 2023), we identified five active components in AG (Table 1), which primarily include D-galactose, L-arabinose, L-rhamnose, D-glucuronic acid, and 4-O-methyl-glucuronic acid. The molecular expressions in Canonical SMILES format were imported into the SwissTargetPrediction database, yielding a total of 255 corresponding targets.

**TABLE 1:** Details of 5 active chemical composition in Arabic Gum (AG) (Ashour et al., 2022; Afoakwah et al., 2023).

Compound name	Molecular function	Canonical SMILES	Content
D-galactose	C_6_H_12_O_6_	C(C1C(C(C(C(O1)O)O)O)O)O	32.5%–35.0%
L-arabinose	C_5_H_10_O_5_	C1C(C(C(C(O1)O)O)O)O	31.7%–53.1%
L-rhamnose	C_6_H_12_O_5_	CC1C(C(C(C(O1)O)O)O)O	2.7%–16.3%
D-glucuronic acid	C_6_H_10_O_7_	C1(C(C(OC(C1O)O)C(=O)O)O)O	5.3%–14.0%
4-O-methyl-glucuronic acid	C_7_H_12_O_7_	COC1C(C(C(OC1C(=O)O)O)O)O	0.8%–5.2%

**FIGURE 1:**
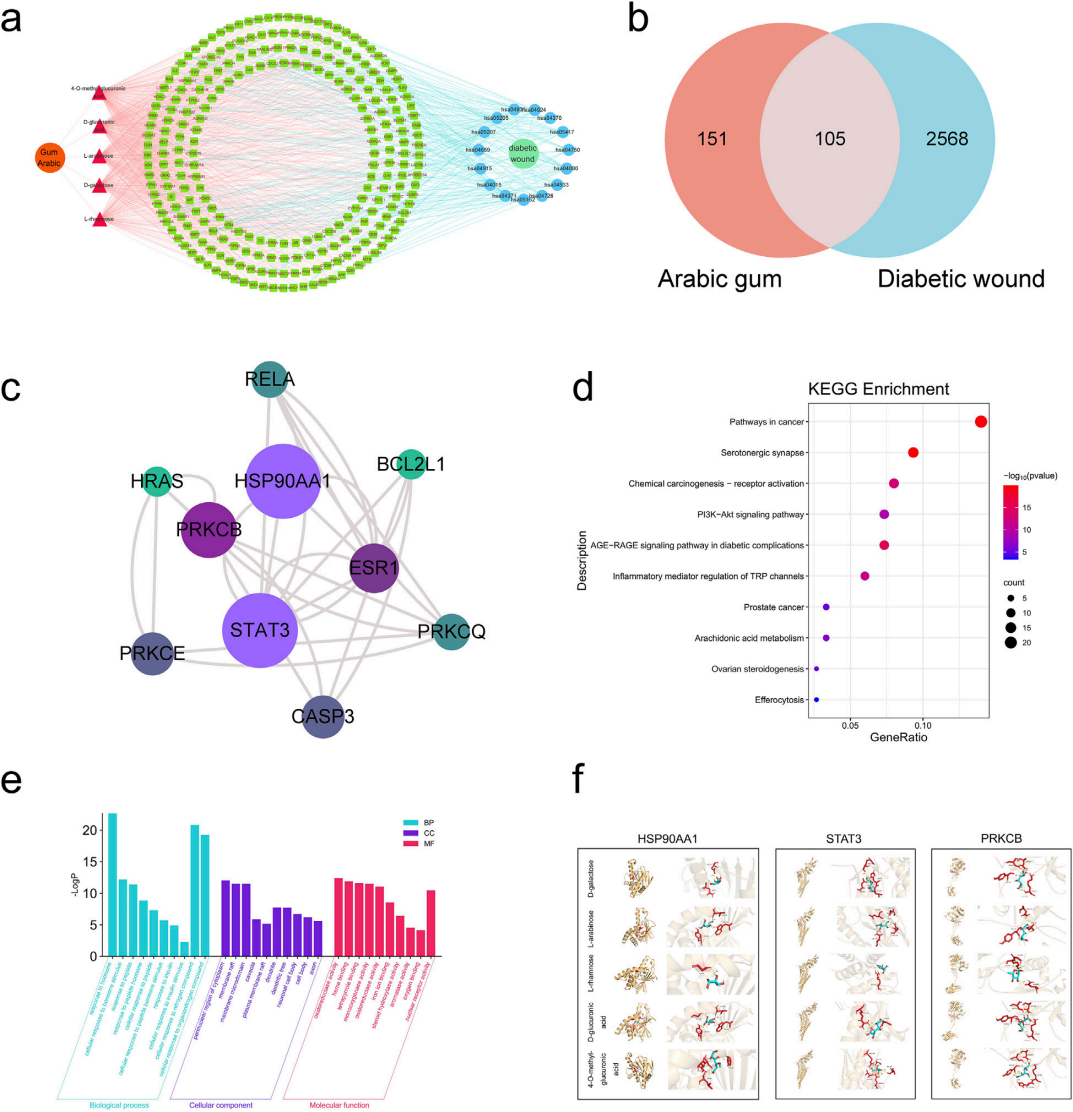
Network pharmacology-based analysis. **(A)** AG-components-targets-pathways-diabetic wound; **(B)** Venn diagram of active ingredients and disease targets; **(C)** The top 10 core targets among the intersecting genes; **(D)** GO analysis of the intersecting genes between AG and diabetic wound; **(E)** KEGG analysis of the intersecting genes between AG and diabetic wound; **(F)** The combinations of pivotal compounds and protein targets. In the figure, the red sections represent the drug molecules, while the yellow portions depict the protein structures. The middle image shows the overall binding mode between these two, and the right-side image is an enlarged view of the molecular and protein interactions, with dashed lines indicating hydrogen bonds.

The AG active component targets and diabetic wound targets were imported into the Venny 2.1 website, which yields 105 intersecting targets. Aside from that, a Venn diagram was generated (Figure 1B). The 105 intersecting targets were uploaded to the STRING platform. Subsequently, a PPI (protein-protein interaction) network was generated (Supplementary Figure S1) subsequent to the removal of disconnected nodes. Topological analysis of the resulting PPI network was conducted by utilizing the CentiScaPe 2.2 plugin in Cytoscape 3.8.2. Ultimately, 10 core targets were identified (Figure 1C). It was observed that AG primarily acts on diabetic wound-related targets such as HSP90AA1, STAT3, PRKCB, and ESR1.

On the basis of the lowest P-values, the top 10 biological processes (BP) include cellular responses to hormone and hormone stimuli, cellular responses to peptide, peptide hormone, and peptide hormone stimuli, cellular responses to insulin and insulin stimuli, and cellular responses to nitrogen compound. The top 5 cellular components (CC) are the perinuclear region of the cytoplasm, membrane rafts, membrane microdomain, caveola and plasma membrane rafts. The top 5 molecular functions (MF) are oxidoreductase activity (acting on paired donors, with incorporation or reduction of molecular oxygen), heme binding, tetrapyrrole binding, monooxygenase activity, and oxidoreductase activity. (The bar chart for the top 10 is depicted in Figure 1D). KEGG pathway analysis identified 150 signaling pathways (P < 0.05). The top 5 pathways are: pathways in cancer, serotonergic synapse, chemical carcinogenesis-receptor activation, PI3K-Akt signaling pathway, and AGEs-RAGE signaling pathway in diabetic complications (the bubble chart for the top 10 is illustrated in Figure 1E).

As universally acknowledged, a docking score of <0 kcal/mol suggests that a compound and target can bind spontaneously, with scores <−4.25 kcal/mol indicating desirable docking affinity, and scores <−7 kcal/mol being indicative of strong binding affinity (Gaillard, 2018). For this reason, we obtained the crystal structures of three target proteins from the PDB database and used BDSV to predict the locations of the binding pockets and the dimensions of the grid boxes (Table 2). The key targets from the intersecting genes were ranked by degree value, while the top three key targets, along with their corresponding AG active components, were selected for molecular docking. The three sets of target proteins and compound molecules were imported into AutoDock Vina, and the binding affinity values for the best docking poses were calculated (Table 3). All values were below −4.25 kcal/mol, suggesting satisfactory docking affinity. As a consequence, molecular docking supports the therapeutic potential of AG in the treatment of diabetic wounds (Specific combinations are displayed in Figure 1F).

**TABLE 2:** Details of the top 3 targets related to AG-Diabetic wounds, as identified from the Protein Data Bank (PDB) database.

Target gene	PDB ID	Protein pocket coordinates	Grid box size
HSP90AA1	2QG2	x = 1.043, y = 31.279, z = 28.196	x = 46.0, y = 42.0, z = 46.0
STAT3	6NJS	x = −4.846, y = 19.708, z = 24.77	x = 88.0, y = 118.0, z = 92.0
PRKCB	3PFQ	x = −59.974, y = 5.387, z = −15.979	x = 86.0, y = 112.0, z = 76.0

**TABLE 3:** Binding affinity values of the optimal docking conformations of 3 sets of target proteins and compound molecules.

Protein affinity kcal/mol compound	2QG2	6NJS	3PFQ
D-galactose	−4.6	−4.3	−5.3
L-arabinose	−5.0	−4.6	−5.1
L-rhamnose	−5.5	−5.0	−5.5
D-glucuronic acid	−5.7	−4.7	−5.8
4-O-methyl-glucuronic acid	−4.7	−5.0	−5.5

### 3.2 *In vitro* cellular functional assays

#### 3.2.1 Biocompatibility of AG

**FIGURE 2:**
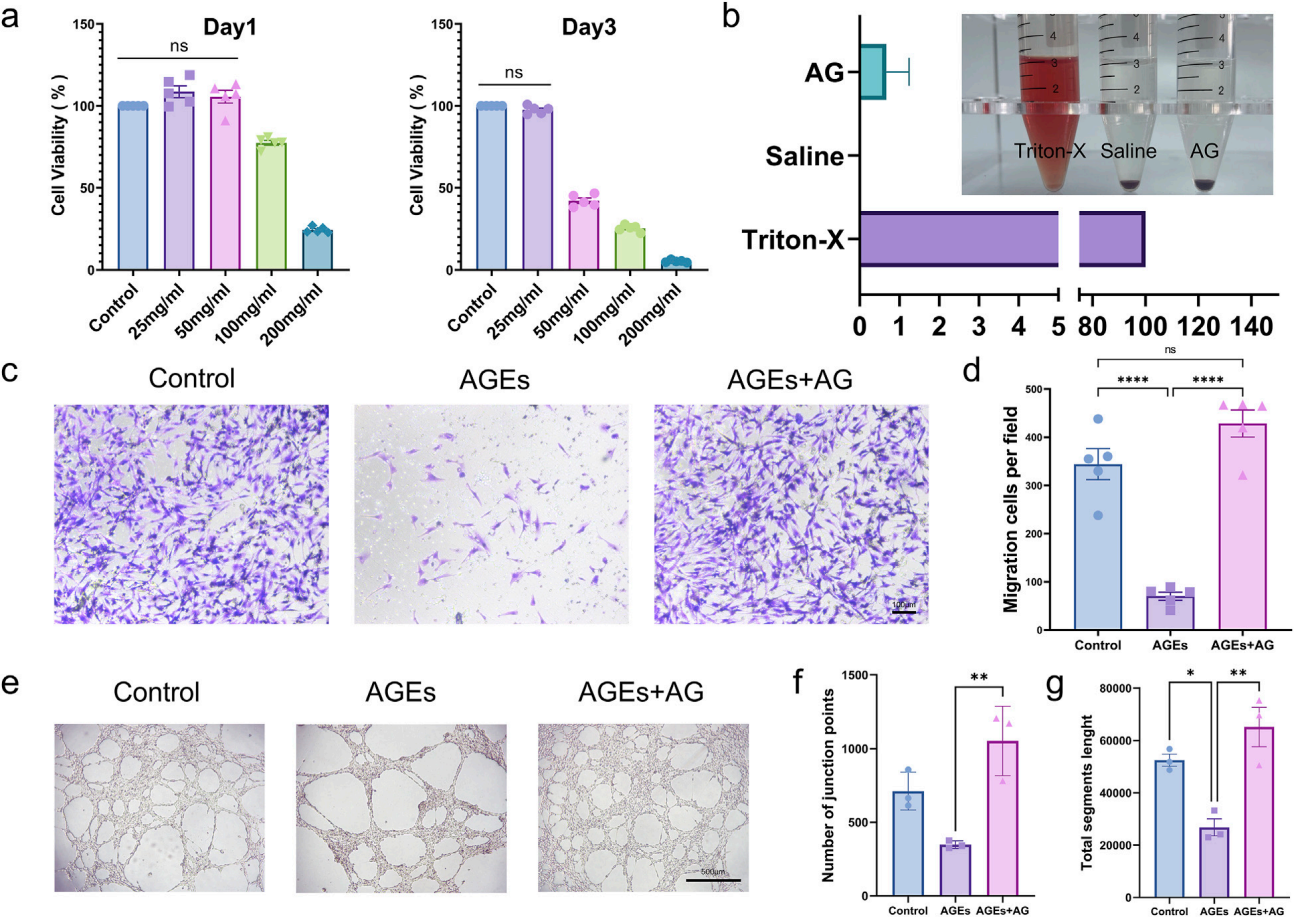
*In vitro* cellular functional assays. **(A)** Cell viability of fibroblasts treated with AG at different time points (Day 1 and Day 3) for *in vitro* proliferation; **(B)** Percentage of hemolysis induced by 25 mg/mL AG; **(C)** Representative images showing the effects of the control group, AGEs group, and AGEs + AG group on HSFs migration; **(D)** Quantification of HSFs migration; **(E)** Representative images showing the effects of the control group, AGEs group, and AGEs + AG group on HUVECs tube formation; **(F)** Quantification of the number of junction points in HUVECs tube formation; **(G)** Quantification of the total segments length in HUVECs tube formation.

In some sense, biocompatibility is reckoned as a predominant requirement for biomaterials in wound dressings (Siavash and Noursina, 2023). To determine the effects of AG on human cells, cell viability of HSFs treated with dissimilar concentrations of AG was measured by employing the CCK-8 assay (Figure 2A). As conspicuously demonstrated by the experimental findings, at an AG concentration of 20 mg/mL, the cell viability was 108.6% ± 8.1%. This concentration was selected for subsequent experiments. Aside from that, a hemolysis assay was performed (Figure 2B) to assess the hemocompatibility of AG. The experimental results display that AG has similar hemocompatibility to the negative control, suggesting its suitability for human use. The half-maximal inhibitory concentrations (IC50) of AG at 24 and 48 h were calculated to be 144.6 mg/mL and 41.49 mg/mL, separately (Supplementary Figure S2).

Whether cells can efficiently and quickly migrate to the center of a wound is one of the decisive factors affecting wound healing (Martin and Nunan, 2015). On that account, a Transwell cell migration assay was conducted to further look into the effect of AG on HSFs migration. AGEs substantially inhibited HSFs migration in contrast to the control group, while the addition of AG effectively reversed this inhibition (Figures 2C, D). Another major factor hindering diabetic wound healing is lessened angiogenesis. On this basis, so a tube formation assay by employing HUVECs was performed to dig into the effect of AG on angiogenesis. The junction points generated in the control, AGEs, and AGEs + AG groups were 711.3 ± 128.7, 347.7 ± 25.7, and 1053 ± 235.9, separately. The total segment lengths were 52,445 ± 4,031, 26,783 ± 5,628, and 65,133 ± 12,975, severally (Figures 2E–G). As these findings suggest, AG can effectively reverse the negative effects of AGEs on cell migration and angiogenesis, even surpassing the control group.

#### 3.2.2 AG regulates the NF-κB pathway to reduce AGEs-Induced activation of the NLRP3 inflammasome

**FIGURE 3:**
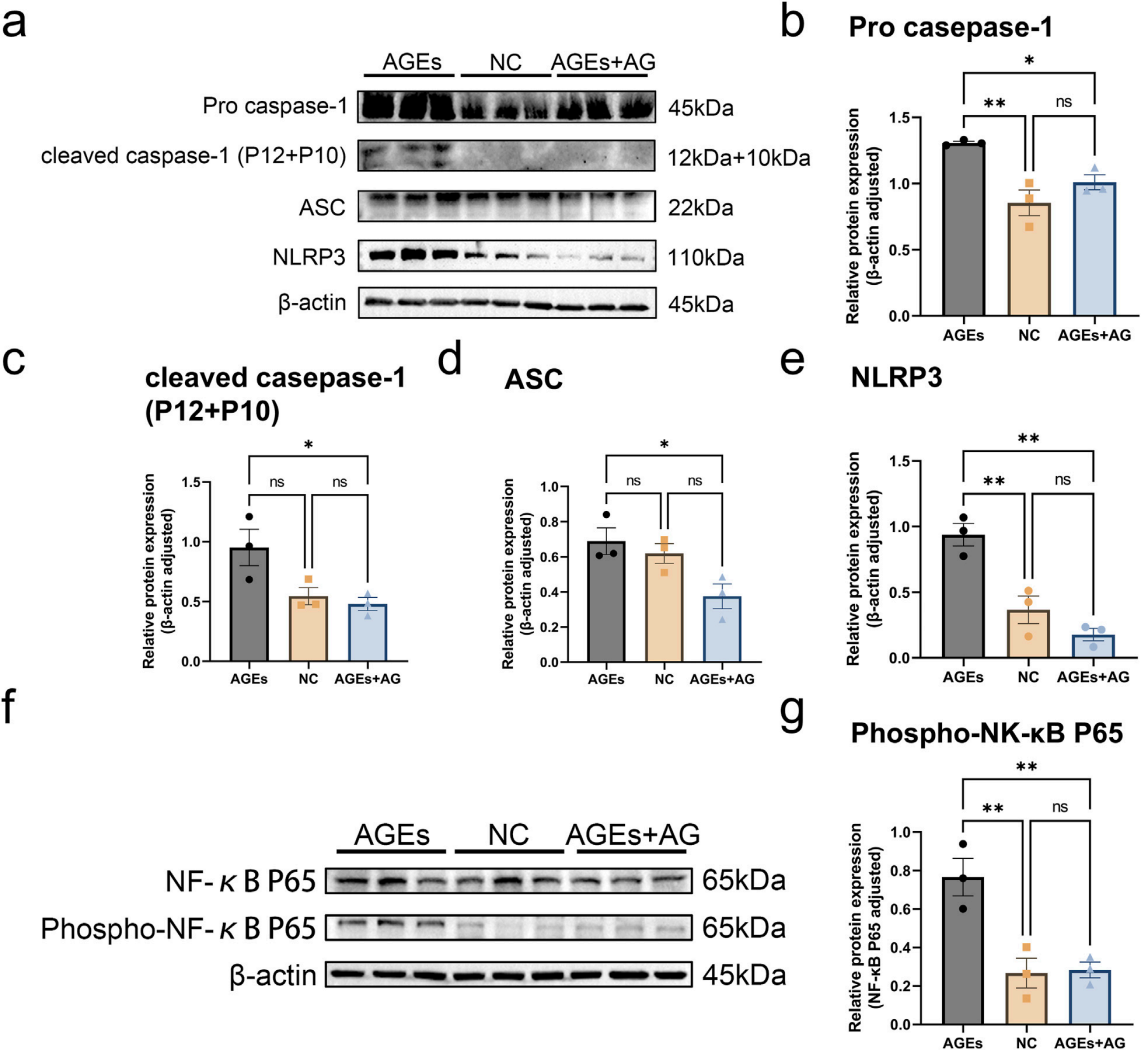
AG regulates the NF-κB pathway to reduce AGEs-induced activation of the NLRP3 inflammasome. **(A)** Representative Western blotting images of Pro caspase-1, cleaved caspase-1 (P12 + P10), ASC, NLRP3 and β-actin; **(B–E)** Quantification of fold-change of: **(B)** Pro caspase-1; **(C)** cleaved caspase-1 (P12 + P10); **(D)** ASC; **(E)** NLRP3; **(F)** Representative Western blotting images of NF-κB p65, Phospho-NF-κB p65 and β-actin; **(G)** Quantification of fold-change of Phospho-NF-κB p65.

As evidently suggested by the research findings, AGEs can mediate the activation of the NLRP3 inflammasome through oxidative stress (Wan et al., 2022). T As a consequence, we delved further into whether AG could inhibit inflammasome activation by measuring key components of the NLRP3 inflammasome, encompassing the NOD-like receptor NLRP3, the adaptor protein ASC, and caspase-1. Treating RAW cells with 300 μg/mL AGEs activated the NLRP3 inflammasome, giving rise to elevated protein levels. In contrast to the AGEs-treated group, the AG-treated group showed a reduction in the protein levels of Pro-caspase-1, ASC, and NLRP3 by approximately 1.3-fold (p < 0.05), 1.8-fold (p < 0.05), and 5.3-fold (p < 0.01), separately, after 24 h (Figures 3A–E).

In an effort to further elucidate the regulatory mechanism of AG on AGEs-induced cellular dysfunction, we probed into Phospho-NF-κB p65, a pivotal regulator in the NF-κB signaling pathway. The expression level of p-p65 was measured by Western blot. In comparison with the control group, treatment with 300 μg/mL AGEs reinforced p65 phosphorylation (p < 0.01), thereby activating the NF-κB pathway. Nevertheless, co-treatment with AG dramatically inhibited the AGEs-induced increase in p-p65 protein expression (Figures 3F, G). As these findings demonstrates, AG regulates the NF-κB pathway to reduce AGEs-induced activation of the NLRP3 inflammasome, thereby exerting a positive effect on the wound healing process.

#### 3.2.3 *In vivo* diabetic wound healing effect of AG

**FIGURE 4:**
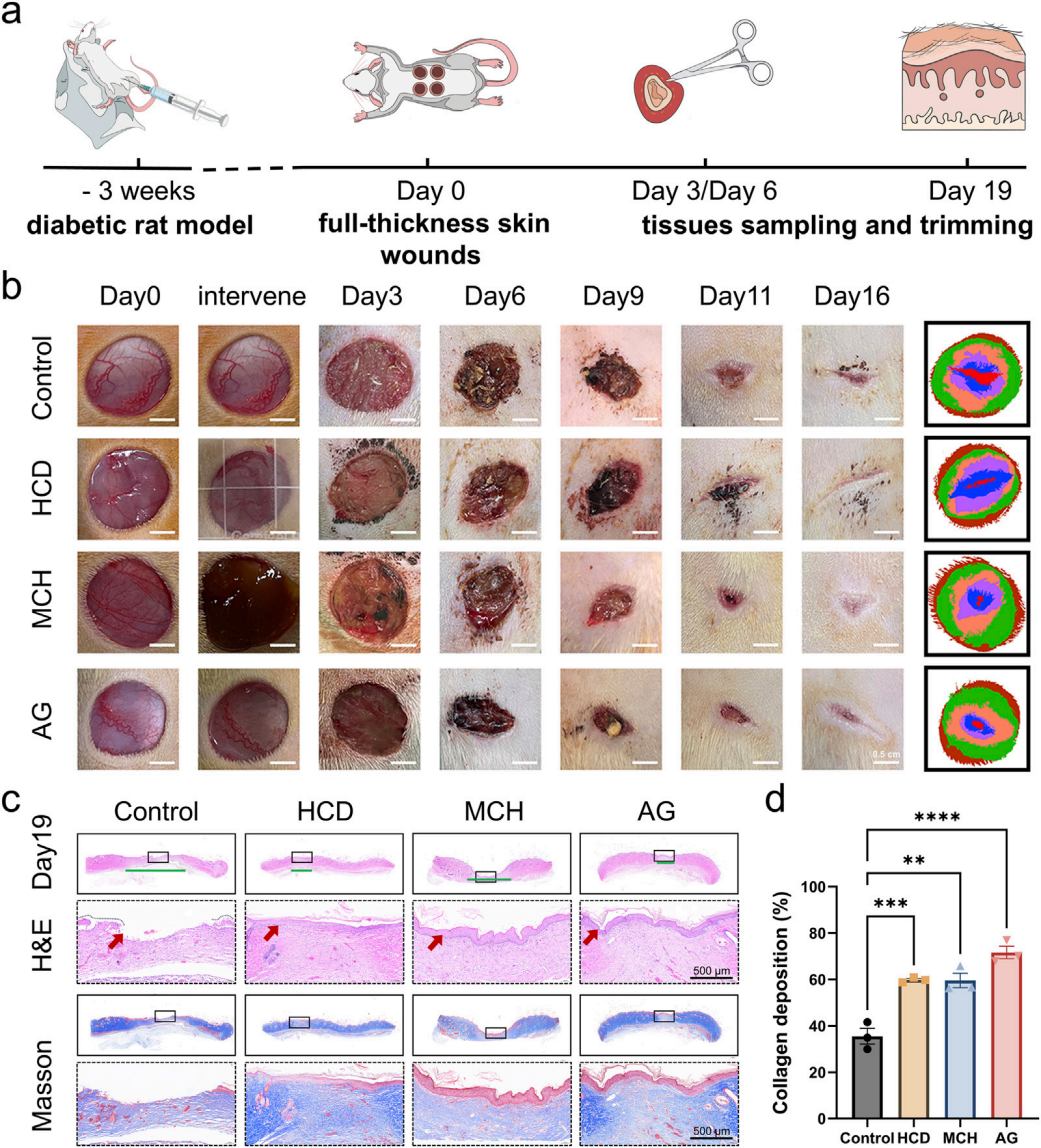
*In vivo* diabetic wound healing effect of AG. **(A)** Schematic diagram of the animal experiment protocol; **(B)** Representative images of wound healing progression in the control, HCD, MCH, and AG groups; **(C)** Representative H&E and Masson’s staining images of wound tissue from different groups on day 19 post-wounding (The green line segment denotes the area of the subcutaneous wound. The red arrow symbolizes the epidermis.); **(D)** Quantification of collagen deposition in wound tissues from different groups on day 19 post-wounding.

The wound healing ability of AG was studied by adopting a full-thickness skin defect model in diabetic rats (Figure 4A). The wound size markedly lessened from day 3 and continued to decrease over time (Figure 4B). In contrast to the control group, the MCH and AG groups exhibited higher degree of wound closure on days 9, 11, and 16 of treatment. By day 16, nearly all wounds were almost fully closed, while lesions in the control group remained visibly noticeable. On day 19, the rats were euthanized, and skin tissue from the wound areas of all four groups was excised for histological evaluation. As revealed by H&E staining, only the control group’s wound tissue lacked a complete and continuous epithelial structure by day 19 (Figure 4C). Nevertheless, the application of AG was bound up with the smallest wound width (Figure 4C), demonstrating a more rapid healing process by AG treatment. As demonstrated by masson’s trichrome staining, the collagen deposition rate in the AG group reached 71.6% ± 4.6%, which was not conspicuously dissimilar from the HCD group and MCH group, but remarkably different from the control group. This distinction highlights the potential of AG treatment to strengthen collagen fiber deposition, thereby giving rise to ameliorated wound healing and tissue regeneration in the treatment groups.

#### 3.2.4 Histological analysis

**FIGURE 5:**
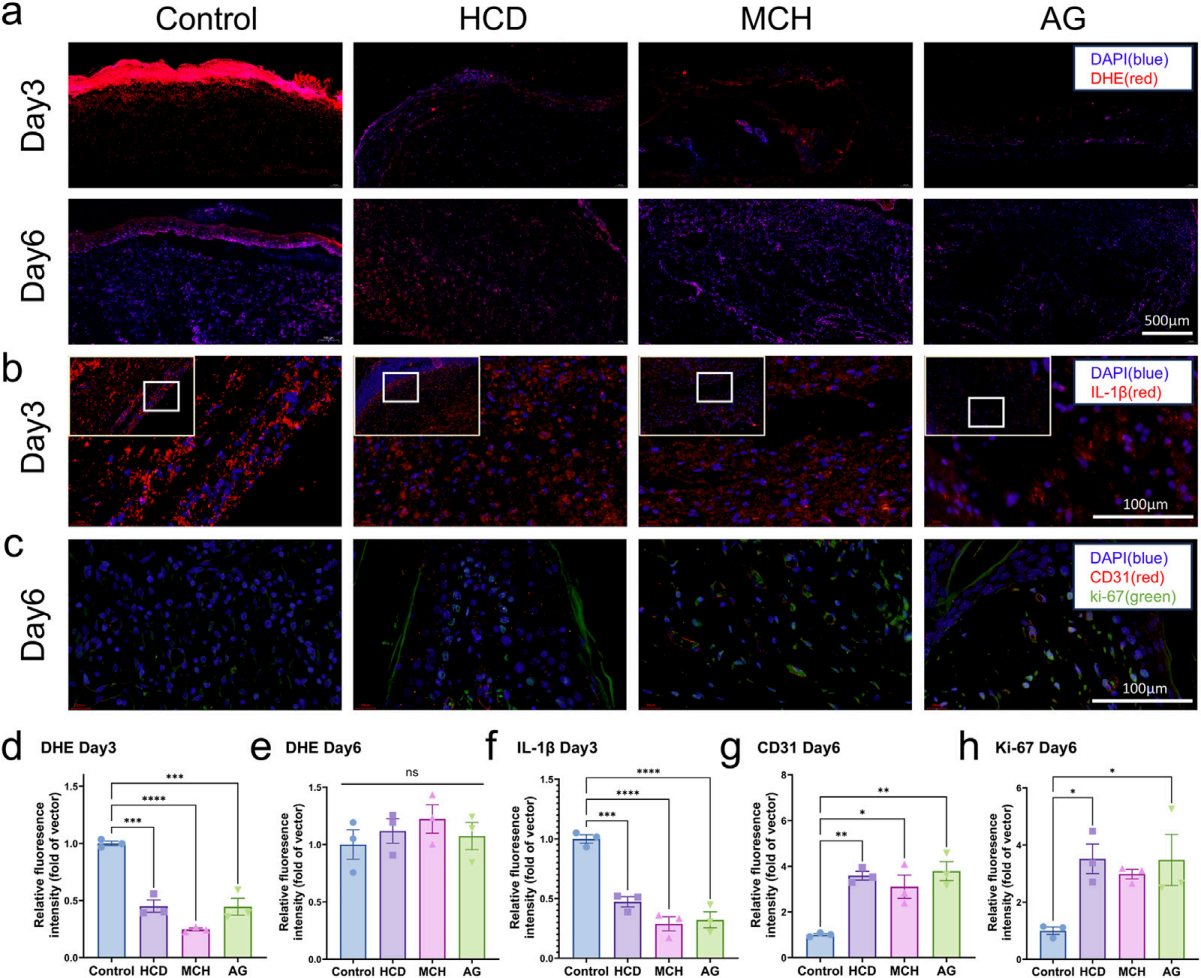
Immunofluorescence staining of skin tissues post diabetic wound healing. **(A)** Representative images of DHE staining on days 3 and 6 post-wounding; **(B)** Representative images of IL-1β staining on day 3 post-wounding; **(C)** Representative images of CD31 and Ki-67 staining on day 6 post-wounding; **(D)** Quantification of DHE staining on day 3 post-wounding; **(E)** Quantification of DHE staining on day 6 post-wounding; **(F)** Quantification of IL-1β staining on day 3 post-wounding; **(G)** Quantification of CD31 staining on day 6 post-wounding; **(H)** Quantification of Ki-67 staining on day 6 post-wounding.

An Immunofluorescence analysis was performed on each group of samples to delve into the effects of AG on ROS generation, inflammatory infiltration, angiogenesis, and cell proliferation in granulation tissue. To start with, we assessed ROS levels in the wound sites of the four groups on days 3 and 6 by employing DHE staining. On day 3, ROS generation was remarkably lowered in the AG group and these two positive control groups in contrast to the control group (p < 0.0001) (Figures 5A, D). By day 6, there were no statistically significant discrepancies between the groups (p > 0.05) (Figures 5A, E). For another, diabetic wounds, being chronic in nature, are featured by prolonged inflammation. For this reason, IL-1β staining was conducted on day 3, while CD68 and CD206 staining was performed on day 6 to assess inflammatory infiltration in the wound area (Dai et al., 2021; Xiao et al., 2024). Quantitative analysis further confirmed that, in comparison with the control group, the AG group substantially lowered the levels of the inflammatory cytokine IL-1β and facilitated the polarization of macrophages from the M1 to the M2 phenotype (Figures 5B, F; Supplementary Figures S3A, C). This also reflects the inflammatory phenotype mediated by the NLRP3 inflammasome.

Angiogenesis is another major challenge in diabetic wound healing. CD31 and Ki-67 staining was conducted on days 6 and 19 to evaluate angiogenic capacity and cell proliferation (Miller et al., 2018; Liu et al., 2023). CD31 and Ki-67 levels were noticeably higher in the AG group in comparison with the control group during the early stages of healing, with a 3.8-fold and 3.5-fold augment, separately (Figures 5C, G, H). In the later stages of healing, only the control group exhibited high CD31 levels, while no conspicuous discrepancies in Ki-67 levels were observed between groups. This may be attributable to the presence of granulation tissue in the control group, which had not fully epithelialized by day 19 (Supplementary Figures S3B, D–E). As the testing results demonstrate, AG suggests exceptional antioxidant, anti-inflammatory, pro-angiogenic, and cell proliferation-promoting effects during the diabetic wound healing process.

**FIGURE 6:**
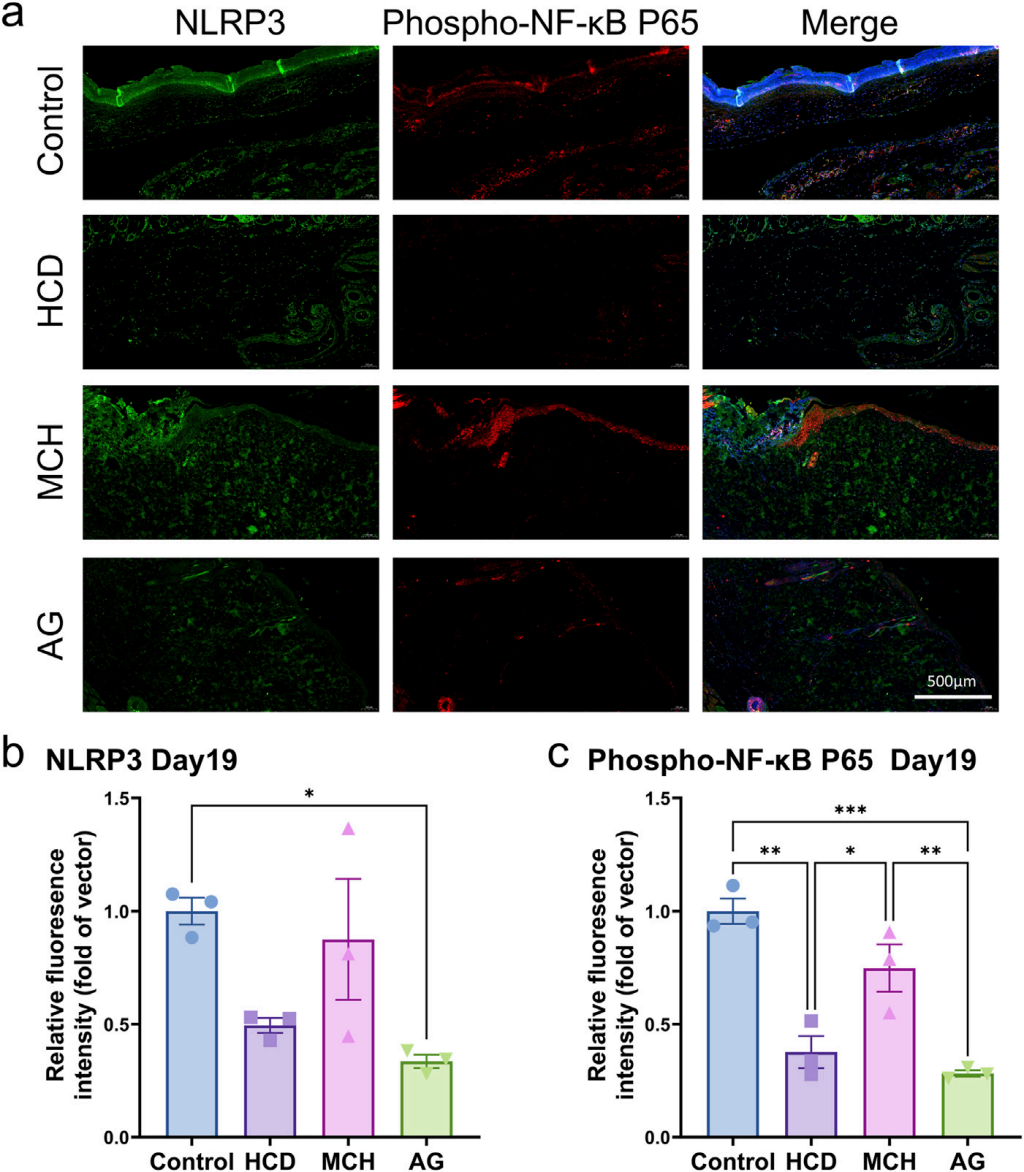
Immunofluorescence staining of skin tissues post diabetic wound healing. **(A)** Representative images of NLRP3 and Phospho-NF-κB p65 staining on day 19 post-wounding; **(B)** Quantification of NLRP3 staining on day 19 post-wounding; **(C)** Quantification of Phospho-NF-κB p65 staining on day 19 post-wounding.

Aside from that, tissue samples on day 19 were stained for NLRP3 and Phospho-NF-κB p65. The AG-treated group revealed dramatically lower levels of NLRP3 (p < 0.05) and Phospho-NF-κB p65 (p < 0.001) in contrast to the control group (Figures 6A–C), which accords with the prior study findings summarized in Western blot analysis. This confirms that AG can lower NF-κB p65 phosphorylation, thereby decreasing NLRP3 inflammasome activation and promoting diabetic wound healing.

## 4 Discussion

Nowadays, there is a deficiency of effective and safe treatment methods for diabetic chronic wounds, which may bring about amputations and severely affect the quality of life of patients (McDermott et al., 2023; Bus et al., 2024). As a result, there is an urgent need for a novel, effective, and safe therapeutic strategy for treating diabetic chronic wounds. This study probed deep into the potential of AG as a therapeutic agent for diabetic chronic wounds through a combination of network pharmacology analysis, molecular docking, and experimental validation both *in vitro* and *in vivo*.

AG is a naturally occurring complex polysaccharide. Studies on diabetic rats have shown that AG exhibits potent therapeutic effects on diabetes (Ibrahim et al., 2023; Keykhaee et al., 2023). In addition to its role in diabetic recovery, AG has been extensively studied in the context of chronic inflammation. Research indicates that AG effectively regulates inflammatory and oxidative biomarkers, contributing to its therapeutic potential (Nemmar et al., 2019; Ali et al., 2020; Gouda and Babiker, 2022). Specifically, AG exhibits significant antioxidant and anti-inflammatory effects, making it a promising agent for cardiovascular protection (Barkeer et al., 2024). In experimental models of ischemia/reperfusion (I/R) injury, AG treatment has been shown to improve cardiac hemodynamics, reduce infarct size, and decrease levels of cardiac enzymes (Gouda and Babiker, 2022). Moreover, AG administration resulted in a marked reduction of pro-inflammatory cytokines, while boosting antioxidant enzyme levels such as superoxide dismutase (SOD) (Gouda and Babiker, 2022).

In our network pharmacology analysis suggested that AG promote diabetic wound healing by regulating AGEs-RAGE signaling pathway, which plays a crucial role in diabetic complications in diabetic complications. Further analysis indicated that the active ingredients include D-galactose, L-arabinose, L-rhamnose, D-glucuronic acid, and 4-O-methyl-glucuronic acid. Additionally, potential target proteins such as HSP90AA1, STAT3, and PRKCB were highlighted as important links in this pathway. HSP90AA1, STAT3, and PRKCB are potential target links (Figure 1). Based on these evidences, the present study focused on the bioactive components of AG to explore their effects on diabetic chronic wound healing and to elucidate the underlying mechanisms that promote wound healing.

In the tissues of diabetic patients, there is a high accumulation of AGEs that can bind to RAGE and activate multiple signaling pathways, such as NF-κB, MAPK, and PI3K-AKT-mTOR (Chen et al., 2016; Li et al., 2025). This activation leads to inflammatory responses, oxidative stress, endothelial dysfunction, and cell apoptosis (Jere et al., 2019; Jin et al., 2024). These events collectively contribute to the pathophysiology of diabetes-related wounds by impairing cellular migration and causing vascular damage, both of which are key factors in the delayed wound healing process. (Morton and Phillips, 2016; Patel et al., 2019). As clearly demonstrated by our findings, AGEs remarkably inhibited the migratory ability of HSFs and the tube-forming capacity of HUVECs in comparison with the control group. Nonetheless, co-treatment with AG reversed these effects and even strengthened them (Figures 2C–G). As these results suggest, AG may offer potential advantages in promoting healing in AGEs-related diabetic wounds.

The NLRP3 inflammasome, which is renowned as a sensor for metabolic and inflammatory signals that induces IL-1β maturation, has captured remarkable academic intention (Kim et al., 2017; Chen et al., 2023). The classical activation of the NLRP3 inflammasome requires an initial signal regulated by NF-κB (Lee et al., 2020; Li W. et al., 2022; Teh et al., 2023). As suggested by our research findings, AG conducts a crucial role in inhibiting the NF-κB pathway by decreasing the phosphorylation of the NF-κB p65 subunit, as demonstrated in our Western blot analyses (Figures 3F, G). This inhibition illustrates that AG may hold back the priming phase essential for the activation of the NLRP3 inflammasome. Considering that the NLRP3 inflammasome functions downstream of NF-κB, we hold the opinion that AG’s influence on NF-κB signaling gives rise to diminished levels of NLRP3 components and pro-inflammatory cytokines.

To further validate its feasibility and underlying mechanisms, a full-thickness wound model on the dorsal area of diabetic rats was employed in this study. we validated how AG conspicuously affects the promotion of wound healing (Figure 4B). The reinforced collagen deposition and elevated expression of angiogenic markers like CD31 and proliferation markers like Ki-67 in AG-treated wounds underscore its anti-inflammatory, antioxidant, pro-angiogenic, and cell-proliferative properties in tissue regeneration (Figures 4D, 5C). Apart from that, our *in vivo* results further substantiate this mechanism, where treatment with AG led to lower concentrations of NLRP3 and IL-1β in wound tissues (Figures 5B, F), aligning with our *in vitro* findings (Figures 3, 6).

**FIGURE 7:**
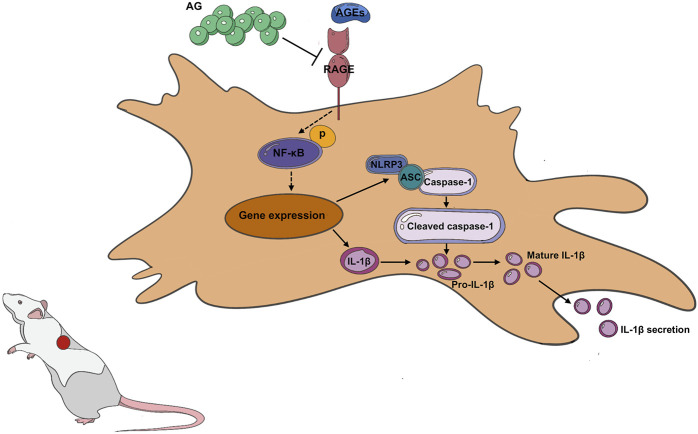
Arabic Gum’s role in promoting diabetic wound healing by modulating the AGEs-NF-κB-NLRP3 axis.

As these findings evidently suggest, AG could reduce chronic inflammation and oxidative stress by disrupting the AGEs-NF-κB-NLRP3 pathway (Figure 7). By intervening in this inflammatory cascade, AG may facilitate a more conducive environment for wound healing under diabetic conditions. It’s pivotal to note that AG’s capability to influence this signaling pathway addresses a paramount component of diabetic wound pathology, which is an ongoing inflammatory condition that obstructs the typical healing process.

While these promising findings are indeed noteworthy, it is crucial to acknowledge several limitations in our research. Above all, the long-term effects and possible side effects of AG treatment were not evaluated in this study. Despite the fact that the FDA generally considers AG safe and it has been utilized as a dietary fiber, its therapeutic application at elevated doses or for extended durations might bring about unexpected negative outcomes. As a consequence, it is essential for future research to dig into the chronic toxicity, immunogenicity, and overall safety of AG concerning diabetic wound healing. Apart from that, this study only examined the dosing from the standpoint of cytotoxicity, and further research is still needed on dose optimization and administration methods. For example, individualized dosage studies could be conducted based on factors such as age, weight, liver function, and kidney function. And research on drug release rates could be explored using hydrogels or microneedle dressings. These aspects will be addressed in future studies by our research team. On top of that, the specific bioactive constituents of AG that induce the observed effects have yet to be fully clarified. For this reason, gaining insight into the distinct roles of individual components could facilitate the creation of more targeted therapies with reinforced efficacy and safety profiles.

## 5 Conclusion

This research findings demonstrate that AG holds potential as a therapeutic agent for improving diabetic wound healing by modulating the AGEs-NF-κB-NLRP3 signaling axis. Nevertheless, addressing the limitations mentioned above is essential for pushing ahead this research. For future endeavors, it’s more preferable to include more extensive experimental validation, exploration of the specific active components of AG, and investigations into the long-term effects and mechanisms in more complex models. Hopefully, such efforts will reinforce our understanding of AG’s role in diabetic wound healing and may drive the development of new therapeutic strategies for this challenging condition.

## Data Availability

The original contributions presented in the study are included in the article/Supplementary Material, further inquiries can be directed to the corresponding authors.

## Abbreviations

AG, Arabic Gum; AGEs, advanced glycation end products; CRF, chronic renal failure; FBS, fetal bovine serum; FDA, Food and Drug Administration; HSFs, human skin fibroblasts; HUVECs, human umbilical vein endothelial cells; PDB, Protein Data Bank; LRR, leucine-rich repeat; NBD, nucleotide-binding domain; PYD, pyrin domain; ROS, reactive oxygen species.
