# Supplementary material for: Pharmacological targets and therapeutic mechanisms of Arabic gum in treating diabetic wounds: insights from network pharmacology and experimental validation
Source: Front Pharmacol. 2025 Feb 20;16:1528880. doi: 10.3389/fphar.2025.1528880 (PMC11882529; doi:10.3389/fphar.2025.1528880)
Supplement: Supplementary file 1 [file DataSheet1.docx]

**Supporting Information**

**Pharmacological Targets and Therapeutic Mechanisms of Arabic Gum in Treating Diabetic Wounds: Insights from Network Pharmacology and Experimental Validation**

Langjie Chai ^1, #^, Danyang Chen ^1, #^, Lili Ye ^1, #^, Pan Peng ^1^, Haijie Wang ^1^, Nouf Al Saleh ^2,3^, Nader S Al-Kenani ^4^, Jia Guo ^1,^ *, Qianqian Li ^1,^ *, Liang Guo ^1,^ *

^1^Department of Plastic Surgery, Zhongnan Hospital of Wuhan University, Wuhan 430071, China

^2^Smart Hybrid Materials Laboratory (SHMs), Division of Physical Science and Engineering, King Abdullah University of Science and Technology (KAUST) Thuwal, 23955-6900, Saudi Arabia.

^3^N. Al Saleh Bioengineering Institute, Health Sector, King Abdul Aziz City for Science and Technology (KACST), Riyadh, 11442, Saudi Arabia.

^4^Department of Orthopedic, Prince Sultan Bin Abdulaziz Humanitarian City, Riyadh, Saudi Arabia.

**^#^**Langjie Chai**,** Danyang Chen **and** Lili Ye **contributed equally to this paper.**

**^*^Corresponding author:**

Guo liang: guolianghbwh@163.com

Qianqian Li: liqian357@163.com

Jia Guo: sindy511@sina.com

**Supplementary figures**


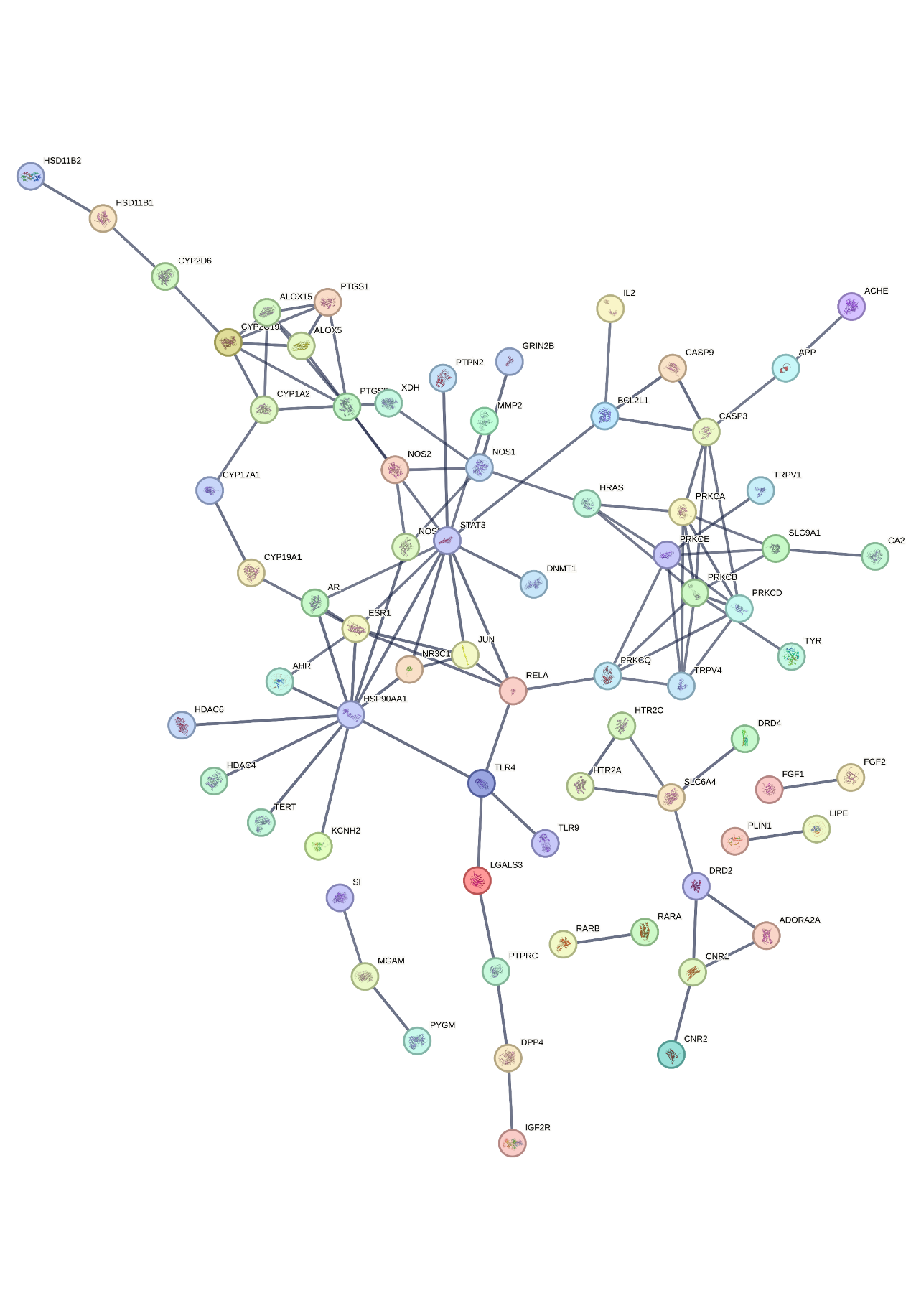
**Fig. S1.** **The protein-protein interaction (PPI) network of the intersecting genes between AG and diabetic wound.**


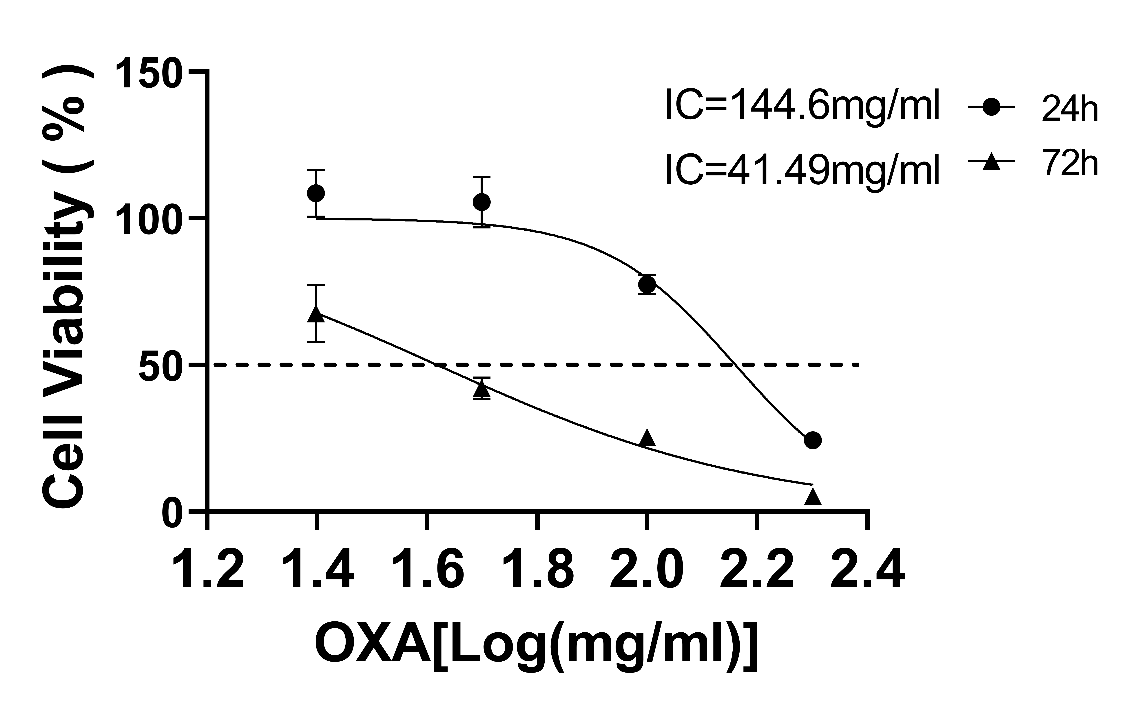


**Fig. S2. The effect of AG on fibroblast proliferation in vitro at different time points (day 1 and day 3), including cell viability and half-maximal inhibitory concentration (IC50).**


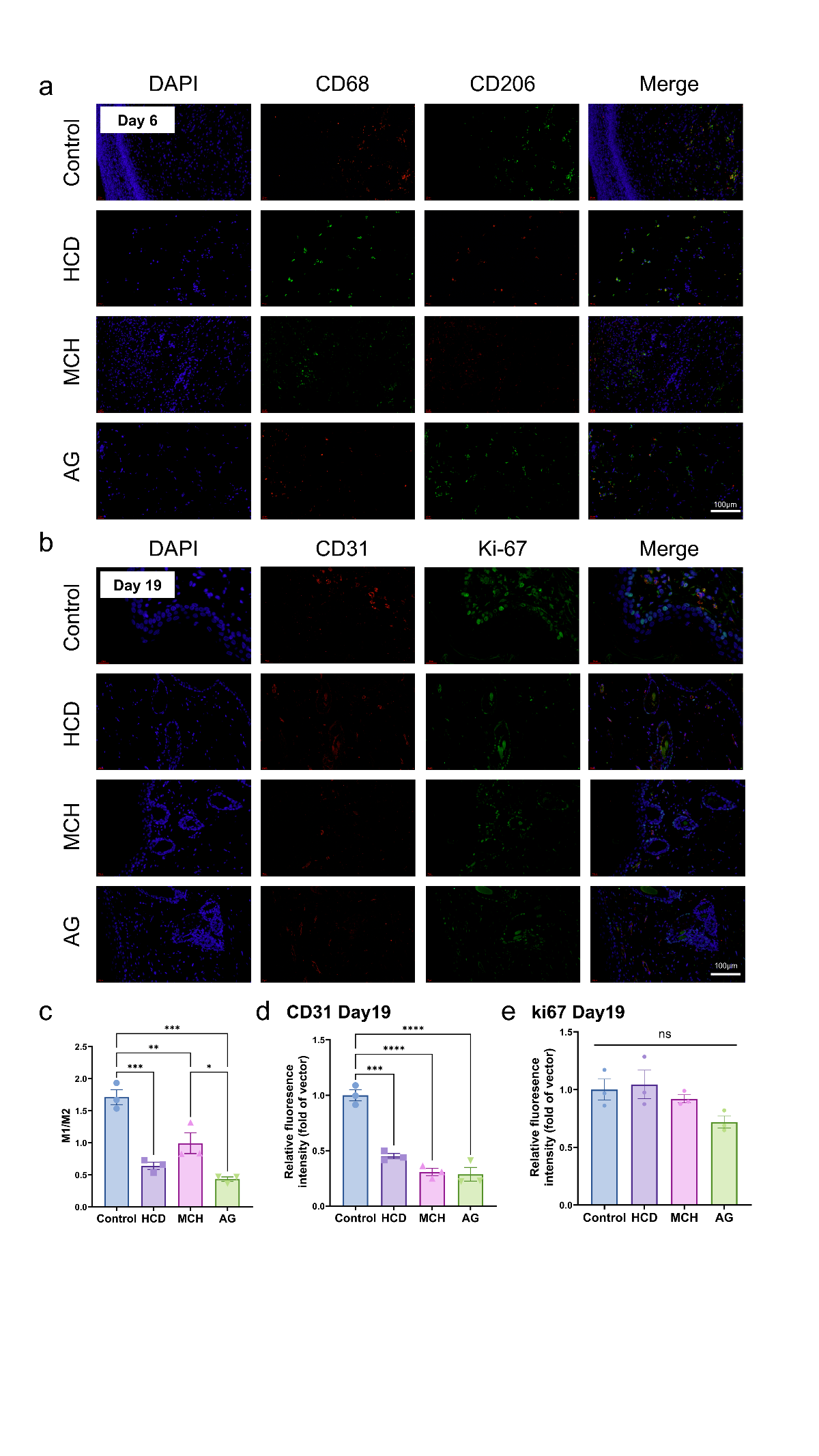


**Fig. S3. Immunofluorescence staining of skin tissues post diabetic wound healing. (a) Representative images of CD68 and CD206 staining on day 6 post-wounding; (b) Representative images of CD31 and Ki-67 staining on day 19 post-wounding; (c) Quantification of M1/M2 macrophages on day 6 post-wounding; (d) Quantification of CD31 staining on day 19 post-wounding; (e) Quantification of Ki-67 staining on day 19 post-wounding.**
